# Low-Frequency Repetitive Transcranial Magnetic Stimulation Ameliorates Cognitive Function and Synaptic Plasticity in APP23/PS45 Mouse Model of Alzheimer’s Disease

**DOI:** 10.3389/fnagi.2017.00292

**Published:** 2017-09-12

**Authors:** Zhilin Huang, Tao Tan, Yehong Du, Long Chen, Min Fu, Yanzhi Yu, Lu Zhang, Weihong Song, Zhifang Dong

**Affiliations:** ^1^Ministry of Education Key Laboratory of Child Development and Disorders, Children’s Hospital of Chongqing Medical University Chongqing, China; ^2^Chongqing Key Laboratory of Translational Medical Research in Cognitive Development and Learning and Memory Disorders, Children’s Hospital of Chongqing Medical University Chongqing, China; ^3^Townsend Family Laboratories, Department of Psychiatry, Brain Research Center, The University of British Columbia Vancouver, BC, Canada

**Keywords:** Alzheimer’s disease, repetitive transcranial magnetic stimulation, spatial learning and memory, long-term potentiation, β-site APP-cleaving enzyme 1

## Abstract

Alzheimer’s disease (AD) is a chronic neurodegenerative disease leading to dementia, which is characterized by progressive memory loss and other cognitive dysfunctions. Recent studies have attested that noninvasive repetitive transcranial magnetic stimulation (rTMS) may help improve cognitive function in patients with AD. However, the majority of these studies have focused on the effects of high-frequency rTMS on cognitive function, and little is known about low-frequency rTMS in AD treatment. Furthermore, the potential mechanisms of rTMS on the improvement of learning and memory also remain poorly understood. In the present study, we reported that severe deficits in spatial learning and memory were observed in APP23/PS45 double transgenic mice, a well known mouse model of AD. Furthermore, these behavioral changes were accompanied by the impairment of long-term potentiation (LTP) in the CA1 region of hippocampus, a brain region vital to spatial learning and memory. More importantly, 2-week low-frequency rTMS treatment markedly reversed the impairment of spatial learning and memory as well as hippocampal CA1 LTP. In addition, low-frequency rTMS dramatically reduced amyloid-β precursor protein (APP) and its C-terminal fragments (CTFs) including C99 and C89, as well as β-site APP-cleaving enzyme 1 (BACE1) in the hippocampus. These results indicate that low-frequency rTMS noninvasively and effectively ameliorates cognitive and synaptic functions in a mouse model of AD, and the potential mechanisms may be attributed to rTMS-induced reduction in Aβ neuropathology.

## Introduction

Alzheimer’s disease (AD) is a neurodegenerative disorder that affects a large number of elderly people and is characterized clinically by progressive loss of memory and decline of multiple cognitive abilities (Nie et al., 2011). Amyloid-β (Aβ) accumulation is considered to play an essential role in AD pathogenesis by resulting in neuritic plaques, synaptic deficit and neuronal death (Koffie et al., 2009). Aβ derives from the amyloid-β precursor protein (APP), which is cleaved by β-secretase and γ-secretase to yield Aβ, and β-site APP-cleaving enzyme 1 (BACE1) is the β-secretase *in vivo* (Sinha et al., 1999; Vassar et al., 1999; Yan et al., 1999; Hussain et al., 2000; Ly et al., 2013). It has been reported that BACE1 and its activity are significantly increased in the brain of AD patients and various transgenic models of AD (Yang et al., 2003; Zhang et al., 2009). Further genetic studies have shown that overexpression of BACE1 at moderate levels increases APP processing and the steady-state level of Aβ (Bodendorf et al., 2002; Harrison et al., 2003; Chiocco et al., 2004). On the contrary, knockdown or knockout BACE1 expression reduces, even abolishes Aβ generation (Cai et al., 2001; Luo et al., 2001; Roberds et al., 2001; Kao et al., 2004). These observations suggest that an increase in BACE1 expression may contribute to the pathogenesis of AD.

Repetitive transcranial magnetic stimulation (rTMS), a painless and non-invasive method to deliver magnetic stimuli into the brain through the intact scalp, has been widely used in psychiatry, neurology as well as other clinical specialties since the 1980s (Barker et al., 1985; Barker, 1994). It has been reported that rTMS is able to modulate synaptic plasticity via changing the excitability of neurons in specific brain regions or the whole brain in a direct or indirect manner (Miniussi and Rossini, 2011). It is widely accepted that activity-dependent synaptic plasticity including long-term potentiation (LTP) and long-term depression (LTD) in the hippocampus, is the cellular mechanism underlying certain types of learning and memory (Bliss and Collingridge, 1993; Malenka and Nicoll, 1999). It is, however, well documented that LTP in the hippocampal CA1 region is dramatically impaired whereas LTD is significantly facilitated in animal models of AD (Nalbantoglu et al., 1997; Chapman et al., 1999; Shankar et al., 2008) and AD patients (Koch et al., 2012). Therefore, it is reasonable to propose that rTMS may modulate synaptic plasticity and subsequently alleviate memory deficits during AD development. Indeed, recent studies have shown that rTMS treatment alone or combined with cognitive training effectively improve cognitive function in AD patients, such as naming and language performance (Cotelli et al., 2008, 2011; Rabey et al., 2013). However, most of previous studies have focused on the effects of high-frequency rTMS on cognitive function in AD, and the role of low-frequency rTMS in the treatment of AD has not been extensively investigated. In addition, little is known about the cellular and molecular mechanism underlying the amelioration of AD symptoms after rTMS treatment.

In the present study, we wanted to determine whether low-frequency rTMS can improve spatial learning and memory in APP23/PS45 double transgenic mouse model of AD. At the same time, we further explored the influence of rTMS on hippocampal LTP and the pathological changes of AD including neuritic plaques, APP processing and BACE1 expression.

## Materials and Methods

### Animals

SPF grade C57/BL6 (wild type, WT) and APP23/PS45 double mutant transgenic mice (1.5-month old) were selected. All mice were housed in plastic cages in a temperature-controlled (21°C) colony room on a 12 h light/12 h dark cycle, and all electrophysiological and behavioral experiments were conducted during the light cycle. Food and water were available *ad libitum*. APP23 transgenic mice carry human APP751 cDNA with the Swedish double mutation at positions 670/671 (KM→NL) under control of the murine Thy-1.2 expression cassette. PS45 transgenic mice carry human presenilin-1 cDNA with the M146V mutation. The genotype of the mice was confirmed by PCR using DNA from tail tissues (Dong et al., 2015). All procedures were performed in accordance with Chongqing Science and Technology Commission guidelines for animal research and approved by the Chongqing Medical University Animal Care Committee.

### Low-Frequency rTMS Treatment

Both WT and AD mice are randomly divided into two subgroups: WT, WT+rTMS, AD and AD+rTMS. Both AD+rTMS and WT+rTMS groups were treated with one session of low-frequency rTMS daily (between 14:00 and 17:00) for 14 consecutive days (from postnatal day 45 to 58). Similar to our previous report (Tan et al., 2013), highly focusing magnetic-electric stimulator (CCY-III, Wuhan Yiruide Medical Equipment Co., LTD., Wuhan, China) with a round coil (6.5 cm diameter) was held centered tangentially to the center of exposed head of the mouse which was fixed in a suitable cloth sleeve. The pattern of one session rTMS consisted of 20 burst trains, each train contained 30 pulses at 1 Hz with 2-s inter-train intervals, in total 600 stimuli and the pulse width was 70 μs. Stimulation intensity was presented 100% of average resting motor threshold as determined by visual inspection of bilateral forelimb movement in a preliminary experiment in anesthetized mice as described previously (Gersner et al., 2011). The sham group mice were treated similarly to the rTMS group by the reverse side of the coil, but were separated from the head using a 3 cm plastic spacer cube.

### Water Maze Task

To test the hippocampal-based spatial memory, 29 WT (16 for sham treatment and 13 for rTMS treatment) and 28 AD (15 for sham treatment and 13 for rTMS treatment) mice were used to perform the Morris water maze task as described previously (Dong et al., 2015). In brief, after daily treatment with low-frequency rTMS (1 Hz) for 2 weeks, mice (aged 3 months) were subjected to water maze test. The maze consists of a round stainless steel pool (150 cm in diameter), which filled with water mixed with opaque white paint (23 ± 1°C). The pool was surrounded by light blue curtains, and 3 remote visual cues were fixed on the curtains. A CCD camera is suspended right on the pool center to record the animal’s swimming path, and the video output was digitized by the Any-maze tracking system (Stoelting). The pool was artificially divided into four quadrants: NE, NW, SW and SE. The Morris water maze test includes spatial training and probe test. Twenty-four hours before spatial training, the animals were allowed to swim freely in the maze for 120 s adaptation. The spatial learning task was tested four trials per day for five consecutive days. In each trial, mice were placed in the water facing the pool wall from four starting positions (NE, NW, SW, SE), to find the hidden platform (7.5 cm in diameter, located in the SW quadrant), which is submerged in the water at a depth of 1 cm. In each trial, the mice were allowed to swim to find the hidden platform, and then stayed on the platform for 20 s before returning to a cage. Mice failed to find the hidden platform within 120 s were then led to the platform, and stayed for 20 s. Twenty-four hours after the final training trial, mice were returned to the pool from a novel drop point with the hidden platform absent for 120 s, and their swim path was recorded to analysis their spatial memory performance. To exclude the influence of sensorimotor function on learning and memory, the swimming speed was monitored and calculated by escape distance and latency by using the equation: swimming speed = distance/latency.

### Electrophysiology *In Vitro*

Nine WT (6 for sham treatment and 3 for rTMS treatment) and 12 AD (7 for sham treatment and 5 for rTMS treatment) mice at age of 3 months were deeply anesthetized with urethane (1.5 g/kg, i.p.) and transcardially perfused with artificial cerebral spinal fluid (ACSF; in mM: NaCl 124, KCl 2.8, NaH_2_PO_4_.H_2_O 1.25, CaCl_2_ 2.0, MgSO_4_ 1.2, Na-vitamin C 0.4, NaHCO_3_ 26, Na-lactate 2.0, Na-pyruvate 2.0 and D-glucose 10.0, pH = 7.4) prior to decapitation as described previously (Peng et al., 2016). The brain was rapidly dissected and placed in ice-cold ACSF. Hippocampal slices (400 μm) were coronally sectioned with a vibratome (VT1200S, Leica Microsystems, Bannockburn, IL, USA) and then were incubated in ACSF for 2 h at 35°C. A bipolar stimulating electrode was placed at the Schaffer collaterals of dorsal hippocampus CA3 pyramidal neurons, and a recording pipette filled with ASCF was placed at the ipsilateral striatum radiatum of the hippocampal CA1 area. After a 30-min stable baseline, theta burst stimulation (TBS) was given to induce LTP. TBS consisted of two trains of stimuli (at 20 s interval), with each train composed of five bursts (4 pulses at 100 Hz in each burst) at an inter-burst interval of 200 ms. Data acquisition was performed with the PatchMaster v2.73 software (HEKA Electronic, Lambrecht/Pfalz, Germany).

### Immunohistochemistry Staining

After behavioral testing, mice were anesthetized with urethane (1.5 g/kg, i.p., Sigma) and one-half of the brains was immediately frozen for protein extraction. The other half of the brains for immunocytochemical staining was fixed with 4% PFA for 24 h at 4°C. Then cryoprotected with 30% sucrose until the brain sank to the bottom. The brain was coronally sectioned into 20 μm slices. 3% H_2_O_2_ was used to remove residual peroxidase activity for 30 min and rinsed with PBS for 5 min (repeated three times). Then slices were blocked with 5% non-fat milk and incubated overnight with mouse monoclonal 4G8 antibody (1:500) at 4°C to label Aβ. Every sixth slice with the same reference position was mounted onto slides for staining. Plaques were visualized by the avidin-biotin-peroxidase complex (ABC) and DAB (3,3′Diaminobenzidine) method. All visible plaques were counted by microscopy at ×40 magnification in a double-blind manner. The mean plaque count per slice was recorded for each mouse as described previously (Dong et al., 2015).

### Western Blotting

After behavioral testing, the hippocampal tissues from each mouse were collected for western blotting as described previously (Dong et al., 2015; Li et al., 2016). Briefly, the collected hippocampal tissues were lysed on ice in the lysis buffer, and then centrifuged at 14,000 *g* for 10 min at 4°C. Supernatant was collected, and protein concentration was determined by BCA protein assay kit (Thermo Fisher Scientific, Waltham, MA, USA). Equal amounts of protein samples were mixed with 4× sample buffer and boiled at 95°C for 5 min. Proteins were separated on 10% tris-glycine SDS-PAGE or 16% tris-tricine SDS-PAGE and then transferred to immobilon-PTM polyvinylidene fluoride (PVDF) membranes with an electrophoresis apparatus (Bio-Rad, Hercules, CA, USA). The membranes were blocked with 5% non-fat milk in Tris-buffered saline containing 0.1% Tween-20 (TBST) for 1 h at room temperature and then incubated overnight at 4°C with primary antibody. After TBST washing 3 × 5 min, membranes were incubated with horseradish peroxidase-conjugated secondary antibody for 1 h at room temperature. After another three times washing with TBST, the protein was visualized in the Bio-Rad Imager using ECL Western blotting substrate (Pierce). Immunoblotting with anti-β-actin (Sigma; 1:3000) was used to control equal loading and protein quality. Anti-APP antibody (1:1000) C20 was used to detect APP and its C-terminal fragment (CTF) products. Both Anti-APP and Anti-BACE1 antibody (1:1000) were obtained from professor Weihong Song in the University of British Columbia, Vancouver, BC, Canada. The band intensity of each protein was quantified by the Bio-Rad Quantity One software.

### Statistical Analysis

For behavioral and electrophysiological experiments, all data are presented as mean ± SEM. Spatial learning and swimming speed data were analyzed by a two-way ANOVA, with treatment (group) as the between-subjects factor and learning day as the within-subjects factor. All the other data were analyzed by a one-way ANOVA followed by *post hoc* Turkey’s tests, with treatment (group) as the between-subjects factor. For immunohistochemical and immunoblotting assays, all data were analyzed by the Student’s *t*-test and nonparametric Mann-Whitney *U* test, respectively. Significance level was set at *p* < 0.05.

## Results

### Low-Frequency rTMS Treatment Rescues Spatial Memory Deficit in AD Mice

**Figure 1:**
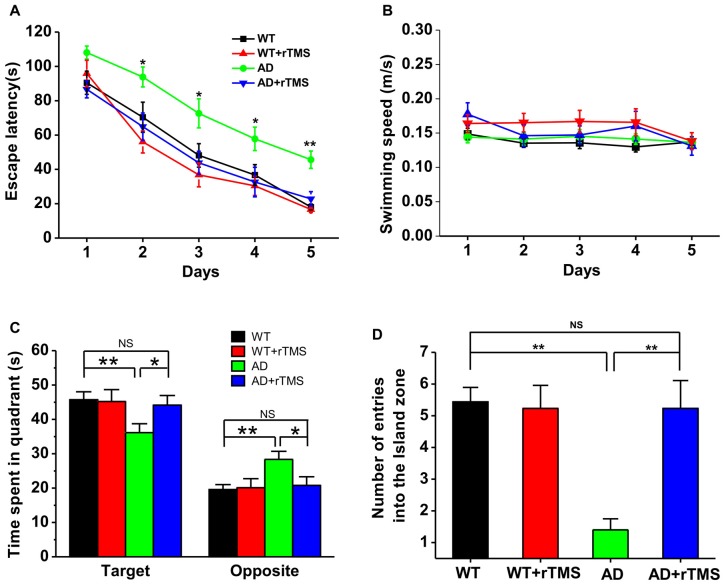
Low-frequency repetitive transcranial magnetic stimulation (rTMS) rescues spatial memory deficits in Alzheimer’s disease (AD) mice. **(A)** The average escape latency to the hidden platform location is plotted for each spatial learning day in the Morris water maze task. **(B)** The similar swimming speed was observed among these groups during spatial learning. **(C,D)** Bar graph showed the time spent in the hidden platform-located quadrant **(C)** and the number of entries into the hidden platform zone **(D)** during the probe test with absence of the hidden platform, which is conducted 24 h after the last learning trial. **p < 0.01, *p < 0.05.

To test the potential rescue effect of rTMS on AD, we first examined the effects of low-frequency rTMS on spatial learning and memory by using a hippocampus-dependent learning and memory task, the Morris water maze, in APP23/PS45 double transgenic AD mice. The AD transgenic mice displayed a significant deficit in spatial learning, as reflected by taking much longer to find the hidden platform than WT control on day 2–5 (WT: *n* = 16, 70.3 ± 8.8 s for day 2, 48.14 ± 6.9 s for day 3, 36.7 ± 6.1 s for day 4, 18.1 ± 1.7 s for day 5; AD: *n* = 15, 93.8 ± 5.8 s for day 2, *p* < 0.05 vs. WT, 72.6 ± 8.4 s for day 3, *p* < 0.05 vs. WT, 57.8 ± 68.8 s for day 4, *p* < 0.05 vs. WT, 45.7 ± 5.1 s for day 5, *p* < 0.01 vs. WT; Figure 1A). Notably, this impairment cannot be attributed to alterations of sensorimotor functions since the swimming speed remained unchanged among these groups (Figure 1B). Importantly, rTMS treatment shortened the escape latency for searching for the hidden platform in AD mice (AD+rTMS: *n* = 13, 64.9 ± 7.9 s for day 2, *p* < 0.05 vs. AD, *p* > 0.05 vs. WT; 43.8 ± 6.6 s for day 3, *p* < 0.05 vs. AD, *p* > 0.05 vs. WT; 32.6 ± 8.6 s for day 4, *p* < 0.05 vs. AD, *p* > 0.05 vs. WT; 22.8 ± 4.2 s for day 5, *p* < 0.01 vs. AD, *p* > 0.05 vs. WT; Figure 1A). A probe test with the platform removed was performed 24 h after the last spatial training trial, to examine long-term spatial memory retrieval. The results revealed that spatial memory retrieval was impaired in AD mice since they spent much less time in the target quadrant in which the platform was previously located (WT: *n* = 16, 45.7 ± 2.3 s in the target quadrant, 19.6 ± 1.5 s in the opposite quadrant; AD: *n* = 15, 36.1 ± 2.6 s in the target quadrant, *p* < 0.01 vs. WT; 28.4 ± 2.3 s in the opposite quadrant, *p* < 0.01 vs. WT; Figure 1C) and reduced the number of entries into hidden platform zone (WT: *n* = 16, 5.4 ± 0.5; AD: *n* = 15, 1.4 ± 0.3, *p* < 0.01 vs. WT; Figure 1D). As expected, rTMS treatment significantly increased the time spent in target quadrant (AD+rTMS: *n* = 13, 44.1 ± 2.8 s in the target quadrant, *p* < 0.05 vs. AD, *p* > 0.05 vs. WT; 20.8 ± 2.5 s in the opposite quadrant, *p* < 0.05 vs. AD, *p* > 0.05 vs. WT; Figure 1C) and the number of entries into the hidden platform zone (AD+rTMS: *n* = 13, 5.2 ± 0.9, *p* < 0.01 vs. AD, *p* > 0.05 vs. WT; Figure 1D). Taken together, these results suggest that low-frequency rTMS treatment can improve spatial learning and memory in AD mice.

### Low-Frequency rTMS Treatment Rescues Impaired Hippocampal LTP in AD Mice

**Figure 2:**
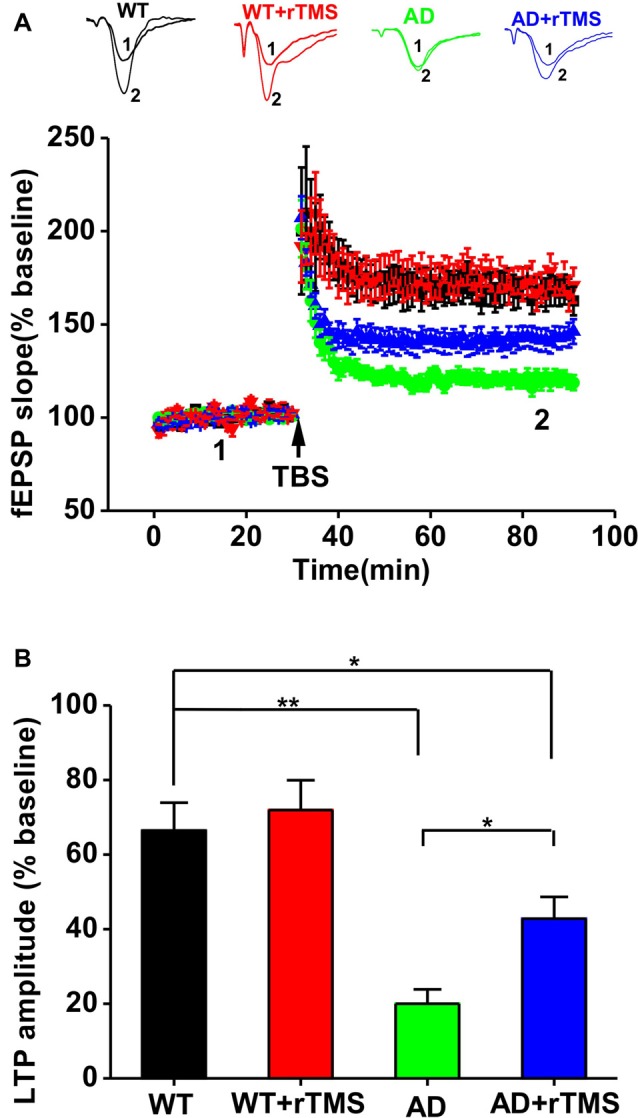
Low-frequency rTMS rescues the impairment of hippocampal long-term potentiation (LTP) in AD mice. **(A)** Representative fEPSP traces and plots of the normalized slopes of the fEPSP 5 min before and 55 min after theta burst stimulation (TBS) delivery. **(B)** Bar graphs of the average percentage changes in the fEPSP slope 55–60 min after TBS delivery. **p < 0.01, *p < 0.05.

We have previously demonstrated that 1 Hz rTMS rescues the impairment of hippocampal LTP in an Aβ-induced toxicity rat model (Tan et al., 2013). In this study, we further investigate the effect of rTMS on hippocampal LTP in APP23/PS45 double transgenic AD mice. Consistent with our recent report (Dong et al., 2015), hippocampal CA1 LTP induced by TBS was impaired in AD mice compared with WT (WT: *n* = 11 slices from six mice, 165.0 ± 7.0%, *p* < 0.01 vs. baseline; AD: *n* = 16 slices from five mice, 118.8 ± 4.0%, *p* < 0.01 vs. baseline, *p* < 0.01 vs. WT; Figures 2A,B). The amplitude of LTP was markedly increased in AD mice treated with rTMS, although it is still smaller than WT (AD+rTMS: *n* = 14 slices from seven mice, 146.2 ± 6.6%, *p* < 0.01 vs. baseline, *p* < 0.05 vs. AD, *p* < 0.05 vs. WT; Figures 2A,B). These results indicate that low-frequency rTMS treatment is partially able to rescue the impairment of hippocampal CA1 LTP in AD mice.

### Low-Frequency rTMS Treatment Reduces AD-Related Neuropathology in AD Mice

**Figure 3:**
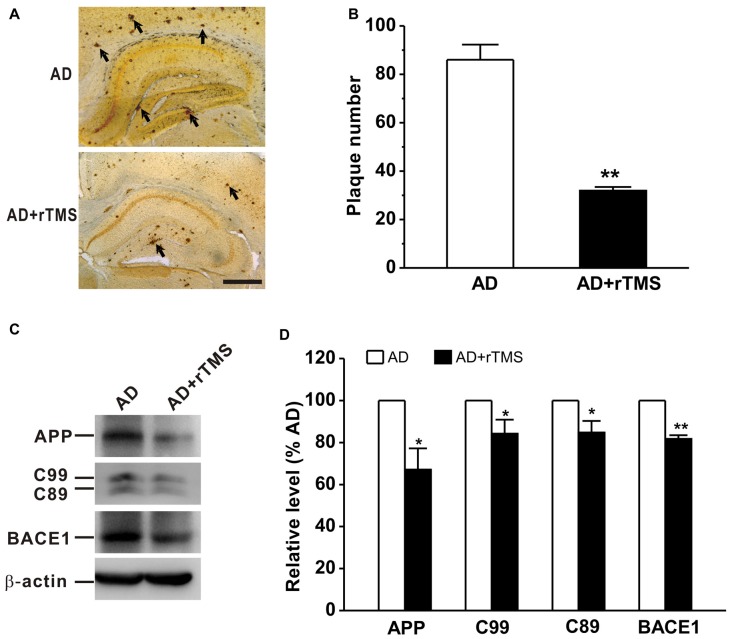
Low frequency-rTMS reduces neuritic plaques, β-site APP-cleaving enzyme 1 (BACE1), amyloid-β precursor protein (APP) and its C-terminal fragments (CTFs) in AD mice. **(A,B)** Low-frequency rTMS decreases neuritic plaque formation (arrows). Scale bar: 500 μm. **(C)** Sequential immunoblotting of total tissue lysates of hippocampal tissues collected from animals after behavioral tests. **(D)** The relative protein level is normalized by the AD group. **p < 0.01, *p < 0.05.

We next want to determine whether rTMS-ameliorated cognitive function and synaptic plasticity in APP23/PS45 mice is attributed to a decrease in Aβ neuropathology such as neuritic plaque formation, APP processing and BACE1 expression. The results showed that the number of plaques was decreased in AD mice treated with rTMS compared with that in AD mice (AD: *n* = 16, 86.0 ± 6.3 plaques; AD+rTMS: *n* = 14, 31.9 ± 1.5 plaques, *p* < 0.01 vs. AD; Figures 3A,B). To further investigate the potential mechanism underlying the reduction of neuritic plaques, we examined the effect of rTMS on APP processing. The level of APP CTFs in the mouse brain tissues was assayed by western blotting analysis (*n* = 5 in each group). The results showed that rTMS treatment significantly decreased the levels of APP (AD+rTMS: 67.1 ± 10.0% relative to AD, *p* < 0.05 vs. AD; Figures 3C,D) and β-secretase-generated C99 (AD+rTMS: 84.2 ± 6.7% relative to AD, *p* < 0.05 vs. AD; Figures 3C,D) and C89 (AD+rTMS: 84.7 ± 5.6% relative to AD, *p* < 0.05 vs. AD; Figures 3C,D) fragments, as well as BACE1 (AD+rTMS: 81.7 ± 1.8% relative to AD, *p* < 0.01 vs. AD; Figures 3C,D), compared with AD mice without treatment with rTMS. The decreased C99 and C89 levels, together with the decreased BACE1 in the brains of the rTMS-treated transgenic mice, indicate that rTMS may inhibit β-secretase cleavage of APP proteins, and subsequently reduced neuritic plaque formation.

## Discussion

In the present study, we confirm that spatial learning and memory as well as hippocampal LTP are significantly impaired in APP23/PS45 double transgenic mice, and demonstrate that low-frequency rTMS treatment alleviates AD-related neuropathology, which may contribute to the amelioration of cognitive function and synaptic plasticity. We have therefore provided evidence that rTMS is an effective and non-invasive brain stimulation method to treat AD and related memory disorders.

So far, no cure has been found for AD. Recently, non-invasive rTMS has been applied to patients with AD and displays beneficial effects on various cognitive functions (Cotelli et al., 2008, 2011; Ahmed et al., 2012; Rabey et al., 2013; Eliasova et al., 2014). Most of these researches have focused on the effects of high-frequency rTMS on cognitive function in AD. However, high-frequency rTMS may occasionally cause some side effects, such as headache and epilepsy or epileptic seizures (Wassermann, 1998; Dobek et al., 2015), whereas no such risk has been reported for low-frequency rTMS to date. Although accumulating evidence have shown that low-frequency rTMS can improve cognitive functions in patients with mood disorders, psychotic disorders, cerebrovascular accident and so on (Lage et al., 2016), it is still under debate whether low-frequency rTMS has beneficial effects on cognitive function in AD patients. Our recent study has shown that low-frequency (1 Hz) rTMS reverses Aβ-induced memory deficits in rats (Tan et al., 2013). A further study reports that 1 Hz rTMS of the right dorsolateral prefrontal cortex (DLPFC) significantly improves the recognition memory performance of mild cognitive impairment (MCI) patients (Turriziani et al., 2012). Consistent with these, we here reported that low-frequency rTMS at 1 Hz dramatically improved spatial learning and memory in APP23/PS45 transgenic AD mice (Figure 1). Nonetheless, contradictory results challenge these findings. For example, Ahmed et al. (2012) have found that low-frequency rTMS was ineffective on cognitive function in AD patients. The exact cause of these discrepancies remains to be determined, but may be at least in part due to the different brain area stimulated, as low-frequency rTMS on the right, but not left DLPFC, significantly improves cognitive function (Turriziani et al., 2012). Notably, we only tested the short-term effects (30 days after rTMS treatment) of low-frequency rTMS on learning and memory in AD mice in the current study. However, AD is a neurodegenerative disorder that will last for a long time. Thus, further experiments on examining the long-term influence of low-frequency rTMS on AD symptoms need to be carried out in the future study.

So far, the mechanism underlying cognitive improvement after rTMS treatment in patients with AD is still poorly understood. Preclinical studies suggested that inhibition of Aβ production by altering APP processing at the β- or γ-secretase site could potentially be avenues for AD drug development (Weggen et al., 2001; Phiel et al., 2003; Li et al., 2006). It has been well documented that fragments of Aβ, abundantly present in the brain, could dramatically interfere with synaptic transmission, leading to impairment of LTP and facilitation of long term depotentiation (LTD) in both animal model of AD (Nalbantoglu et al., 1997; Chapman et al., 1999; Shankar et al., 2008) and patients with AD (Koch et al., 2012). Here, we reported that low-frequency rTMS can reduce BACE1 expression, which leaded to a decrease in APP and its CTFs generation (Figures 3C,D). Subsequently, the decrease of BACE1-mediated APP processing may contribute to the improvement of hippocampal LTP (Figure 2) and spatial learning and memory (Figure 1) after low-frequency rTMS treatment in the present study. In addition, the number of neuritic plaques was decreased after rTMS treatment (Figures 3A,B), indicating an obvious reduction of insoluble Aβ deposits. Therefore, besides the total production of Aβ was decreased by suppressed APP processing (Figures 3C,D), another possibility is that rTMS treatment increases the degradation of insoluble Aβ. Thus, further study examining the degradation of Aβ will help understand the cellular and molecular mechanism underlying the beneficial effects of low-frequency rTMS on AD symptoms. Notably, it is still under debate whether the amount of plaques is correlated with the intensity of observed symptoms (Gruart et al., 2008). However, a growing body of evidence has showed that Aβ could dramatically interfere with synaptic transmission, leading to impairment of LTP and facilitation of LTD (Nalbantoglu et al., 1997; Chapman et al., 1999; Shankar et al., 2008).

Alternatively, it has been well documented that neuronal hyperactivity has been observed in the hippocampus (Palop et al., 2007; Minkeviciene et al., 2009; Harris et al., 2010; Davis et al., 2014; Oyelami et al., 2016) and cerebral cortex (Busche et al., 2008) in AD models. Such hyperactivity could be attributed either to intrinsic hyperexcitability (Minkeviciene et al., 2009; Harris et al., 2010; Davis et al., 2014) or to reduced inhibition (Busche et al., 2008; Oyelami et al., 2016), which may therefore increase the threshold of LTP induction and result in the impairment of LTP in AD mice. Importantly, recent studies have reported that low-frequency rTMS is able to suppress neuronal excitability both in animal and in human (Maeda et al., 2000; Muller et al., 2014). Thus, low-frequency rTMS treatment may ameliorate the impairments of hippocampal LTP in APP23/PS45 mice via regulating the balance of excitatory and inhibitory neuronal activities in the present study (Figure 2). In addition, low-frequency rTMS improves hippocampal LTP through modulating the expression of genes important for synaptic plasticity. For example, rTMS treatment enhances c-Fos and Zif268 expression in different brain areas including hippocampus and cortex (Doi et al., 2001; Aydin-Abidin et al., 2008). Low-frequency rTMS also augments neurotrophin contents such as BDNF and NGF in the hippocampus (Zhang et al., 2007; Wang et al., 2010; Tan et al., 2013).

## Conclusion

Overall, our study demonstrates that low-frequency rTMS can ameliorate the deficits of cognitive and synaptic functions through reducing BACE1 and APP processing in APP23/PS45 double transgenic mice of AD, suggesting that low-frequency rTMS may serve as a highly effective anti-amyloid treatment in AD. However, the molecular mechanisms underlying the beneficial effects of low-frequency rTMS on AD need to be further investigated in the future.

## Author Contributions

ZH, TT, YD, LC, MF, YY and LZ performed the research. ZD designed the research study. TT, WS and ZD contributed essential reagents or tools. ZH and ZD analyzed the data. ZH and ZD wrote the manuscript.

## Conflict of Interest Statement

The authors declare that the research was conducted in the absence of any commercial or financial relationships that could be construed as a potential conflict of interest.
